# Partitioning of amino acids and proteins into decanol using phase transfer agents towards understanding life in non-polar liquids

**DOI:** 10.1038/s41598-019-54322-8

**Published:** 2019-11-28

**Authors:** Brooke Thompson, Kayla Burt, Andrew Lee, Kyle Lingard, Sarah E. Maurer

**Affiliations:** Department of Chemistry and Biochemistry, Central Connecticut State University, New Britain, CT 06050 USA

**Keywords:** Astrobiology, Origin of life, Colloids

## Abstract

Water has many roles in the context of life on Earth, however throughout the universe, other liquids may be able to support the emergence of life. We looked at the ability of amino acids, peptides, a depsipeptide, and proteins to partition into a non-polar decanol phase, with and without the addition of a phase transfer agent. Partitioning evaluated using UV detection, or with HPLC coupled to either charged aerosol detection or ESI-MS. For amino acids and short peptides, phase transfer agents were used to move the biomolecules to the decanol phase, and this transfer was pH dependent. For larger molecules, phase transfer agents did not seem to affect the transfer. Both the depsipetide, valinomycin, and the protein Taq DNA polymerase had solubility in the decanol phase. Additionally, valinomycin appeared to retain its biological ability to bind to potassium ions. These results show that most terrestrial biological molecules are not compatible with non-polar solvents, but it is possible to find and perhaps evolve polymers that are functional in such phases.

## Introduction

Water is often used as a metric to gauge habitability of planets, and while this is a useful measure for human habitation and perhaps all terrestrial life, it is unknown whether water is a necessity for living systems to emerge in general. As we only have one example of life, it is impossible to truly know whether water as a solvent is a necessity, or even beneficial for this process to occur.

Other solvents for life have been discussed previously^1^. While both solids and gases have been proposed as alternative solvents, the diffusion rates in solids and stability/volatility of molecules in gas phases make these phases less appealing to study in a chemistry laboratory, even if there is a possibility of life emerging in them. In liquid phases, generally other polar solvents are suggested, such as brines^2^, ammonia^1^, or sulfuric acid^3^. While these phases have the potential to harbor life, it is unclear if we would find any large-scale liquid pools of these in any of the known planetary bodies.

We do know that there are other liquids within our solar system. Namely there are liquid hydrocarbon lakes on Titan, likely composed mostly of methane and ethane^4^. A recent study estimates that ethane seas may be the most prevalent liquid in the solar system^5^. While there are certainly challenges when working with cryoliquids (e.g., solubility^6^), the abundance of these bodies of liquid demand experimental testing.

Non-polar liquids have previously been considered for protocell formation, generally as an oil droplet in a water phase^7–9^. In these experiments, the bulk oil used was decanol as it easily phase separates from water, does not easily evaporate, and is found in prebiotic sources of organics^10^. While these protocells have been shown to harbor chemical reactions, move, and grow and divide, it is unknown if they can integrate extant biological molecules.

When we think of functional biochemistry, folded polymers generally perform most processes. However, little is known about how important a polar liquid is for the evolution of these functions. There are many membrane proteins that rely on the hydrophobic barrier for function^11,12^, therefore, it is likely that certain proteins or protein-like molecules could serve as an example of functional biopolymers that we would find in non-polar solvents throughout the universe.

One example of a small hydrophobic peptide derivative is valinomycin. This ionophore preferentially transports K^+^ across membranes and is a cyclodepsipeptide composed of both D-and L-alanine^13^. This ionophore is thought to be soluble in the hydrophobic core of the lipid bilayers and uses the carrier method to transport ions. While this molecule is expected to interact strongly with a non-polar phase, most proteins, even membrane proteins, have some interaction with water.

Hydropathy is a measurement that is regularly used for evaluating protein structure to gain insights on folding and other structural elements, additionally the hydropathy of proteins is correlated with cellular location (i.e., membrane or cytosol)^14^. This is done by calculating the grand average hydropathy (GRAVY) score as the average of the hydropathy of all residues within the protein, with positive scores representing higher hydrophobicity (Table 1).

**Table 1:** Hydropathy scores for tested amino acids^14^ and proteins.

Residue Type	Hydrophathy
Phe	2.8
Gly	−0.4
Glu	−3.5
Taq pol^a^	−0.28
BSA^b^	−0.43
Bacteriorhodopsin	+0.72

protein GRAVY calculated for UniProt sequences ^a^P19821 and ^b^P02769.

In the absence of a positive hydropathy, phase transfer agents, like didodecyldimethyl ammonium bromide (DDAB) are often used to increase the permeability of nucleic acid polymers^15^. Additionally, it is known that ion-pairing (like is found in HPLC protocols^16^) can influence the partitioning of peptides and anionic drugs^17,18^.

In this research, we seek to evaluate if biomolecules can use phase transfer agents to increase solubility in the non-polar solvent decanol, and if function can be maintained within that solvent. Using amino acids, peptides, a depsipetide, and proteins we have evaluated the partitioning between a water-decanol phase with and without phase transfer agents (PTAs). These molecules are amphiphiles with a hydrophobic tail and a charged head group. We used decylsulfate (DS) as the anionic transfer agent as negative analogue, to the cationic phase transfer agent DDAB. This co-partitioning allowed for transfer of amino acids and peptides into an oil phase. Proteins and a depsipeptide were also evaluated, but did not rely on the phase transfer agents for partitioning.

## Results

Phase partitioning of biomolecules was undertaken for amino acids and their polymers to determine their solubility with and without the help of charged amphiphiles to serve as phase transfer agents (PTAs). This represents the first time co-solvation has been measured for these systems, and provides a insights into possible mechanisms for biomolecule solubility in oils for understanding non-water liquids and their potential to harbor life.

In generally, the addition of PTAs could improve partitioning of amino acids and small peptide, but did little for proteins. Amino acid and short peptide partitioning in decanol with the addition of PTAs was difficult to predict as it was impacted by at least the charge on the molecules and size, but perhaps other factors such as the biomolecules solubility in water. While the addition of phase transfer agents resulted in phase transfer of charged amino acids that were otherwise insoluble in decanol, there were still low amounts transferred.

The pH was important for the charge on the amino acid: at pH 1.5 the carboxylic acid is neutral, and the amine was positive; at pH 7 the carboxylate was negative and the amine was positive; at pH 10 the carboxylate was negative and the amine was neutral. These charges determined the PTAs used to help counteract the charge on the amino acid. In the absence of phase transfer agents, none of the amino acids entered the decanol phase. To enter the decanol phase, amino acids needed to have a PTA that neutralized the charges and added a hydrophobic tail group.

**Figure 1:**
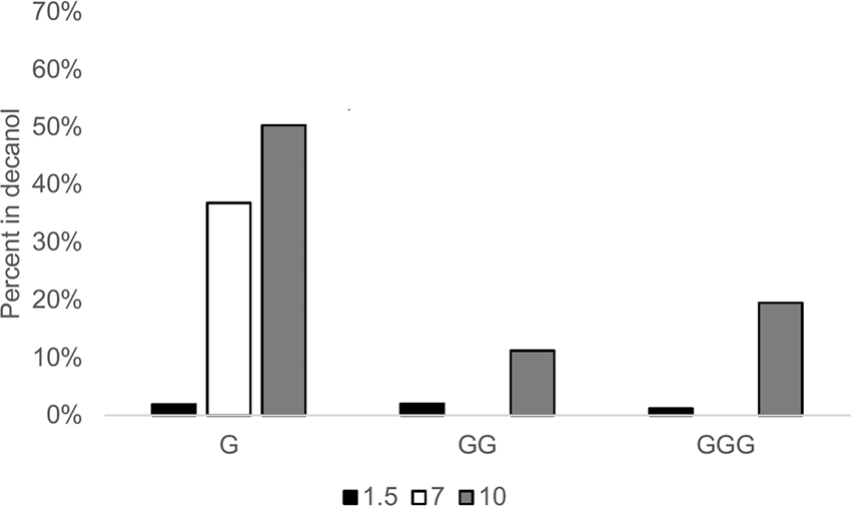
Phase transfer of glycine and its dimer and trimer into decanol at three pHs in the presence of phase transfer agents. All samples were measured in triplicate; deviations between samples were less than 3% with the exception of the GGG pH 10 sample that showed 7% deviation between samples.

Partitioning of mono-, di- and tri-glycine was examined at three pHs: 1.5, 7, and 10 (Fig. 1). Only a very small amount of glycine and its polymers partition at low pH. At high pH all were able to partition with up to 50% glycine transfer for the monomeric form. In general, these peptides tend to have lower solubilities in water than their monomers^19^, however we observed less oil partitioning with di- and tri-glycine in the presence of the phase transfer agent, indicating that water solubility is not a good predictor of PTA-assisted partitioning into decanol. Additionally, at pH 7, no oligoglycine was observed in the decanol phase. Diglycine, and to some extent triglycine, have the ability to form a cyclic structure in water, and perhaps, especially when doubly charged, this could prevent the interaction of the molecule with phase transfer agents.

### Glutamic acid and phenylalanine

**Figure 2:**
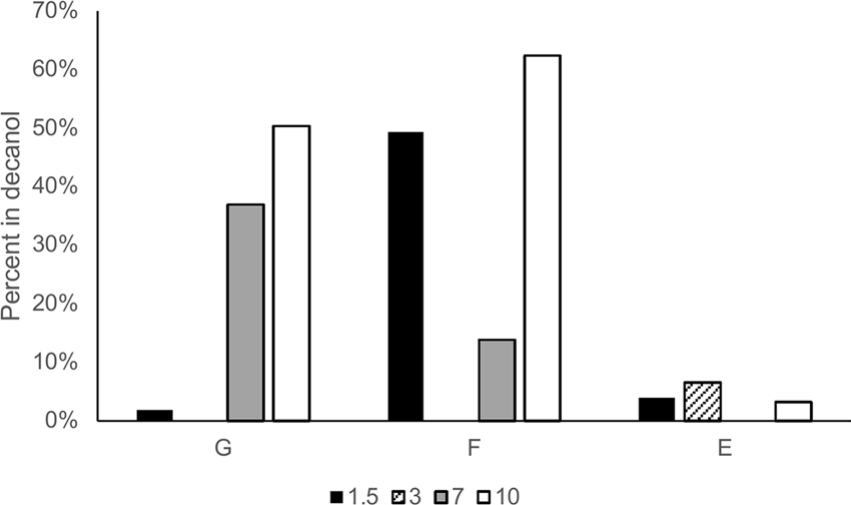
Partitioning of phenylalanine (F) and glutamic acid (E) into decanol with 10 mM PTAs. Only E was tested for partitioning at pH 3, as it has a third pKa for the carboxylate side chain at 4.2. Glycine (G) data is redundant with Fig. 1. The standard deviation all values was less than 3%.

Glutamic acid (E) and phenylalanine (F) were also tested for partitioning into an oil phase with the help of PTAs (Fig. 2). Perhaps unsurprisingly, the addition of a carboxylate group reduces the glutamic acid phase transfer to less than 10% at all pHs, with no transfer seen in the pH 7 (triply charged) samples. Phenylalanine transfers into the decanol phase more readily than glycine at low pH indicating that the addition of the non-polar side chain could increase solubility in decanol. Interestingly, at mid pH of 7, the phenylalanine has a lower abundance in the oil phase than glycine. One possible explanation could be that the bulky phenylalanyl sidechain interferes with the required association of two phase transfer agents, however this has not been fully explored.

**Figure 3:**
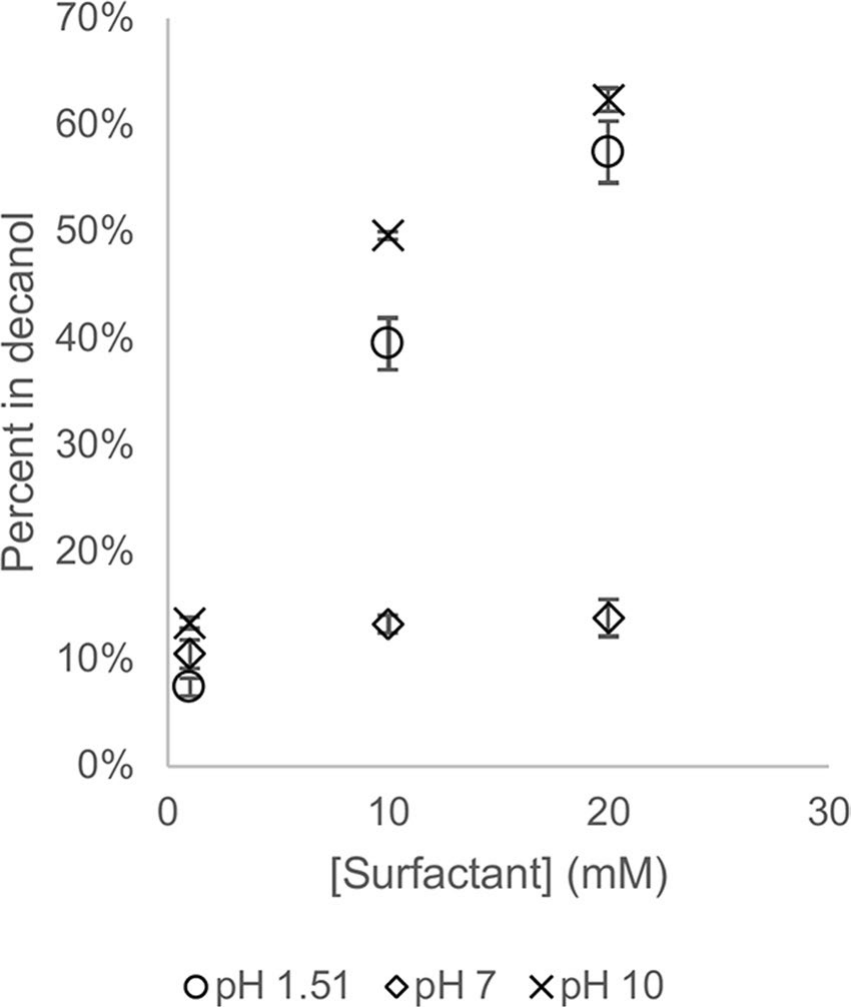
Dependence of partitioning on concentration of phase transfer agents (PTAs). The concentration of phenylalanine in the aqueous layer started at 10 mM and partitioning into decanol was largely controlled by the concentration of DS at pH 1.5 (○), DS and DDAB at pH 7.0 (◇), or DDAB at pH 10.0 (×). At pH 7, 10 mM indicates the sum of surfactants in a 1:1 mole ratio. Error bars represent the standard deviation of three samples.

Phenylalanine transfer into decanol was measured with 10 mM phenylalanine and varying concentrations of PTA at three pHs (Fig. 3). At a 1:10 ratio of PTA to phenylalanine, about 10% of the amino acid entered the decanol phase for all pHs. At a 1:1 ratio of PTA to phenylalanine, in both the high and low pH samples between 40 and 50% of the phenylalanine entered the decanol phase. The pH 7 sample, which is zwitterionic with both a carboxylate and an ammonium group, showed only 15% partitioning. A similar trend was seen at a 2:1 ratio of PTA to phenylalanine, indicating that, especially for the doubly charged phenylalanine, a saturation limit maybe reached.

### Valinomycin

Valinomycin was found to partition into decanol, without PTAs. Ultra-pure Millipore grade (18.2 MΩ) water, 100 mM KCl or 100 mM AuCl were tested as aqueous phases. In all samples all of the valinomycin was found in the decanol phase (see SI for additional details).

These results indicate that it is possible to generate small biopolymer that prefers oil phases. It is likely that other antibiotic peptides, like gramicidins could have similar properties. The transfer of ions into an oil phase could be essential for oil-based life, as transition metals have a large role in redox chemistry, and ions in general can assist with folding and stability of polymers.

### Proteins

**Figure 4:**
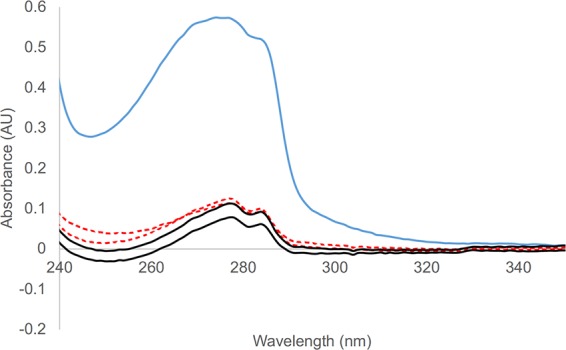
Partitioning of Taq polymerase into decanol. Tpol with (red dashed line) and without (black solid line) 5 mM PTA in decanol are compared to starting water phase (blue solid line).

**Figure 5:**
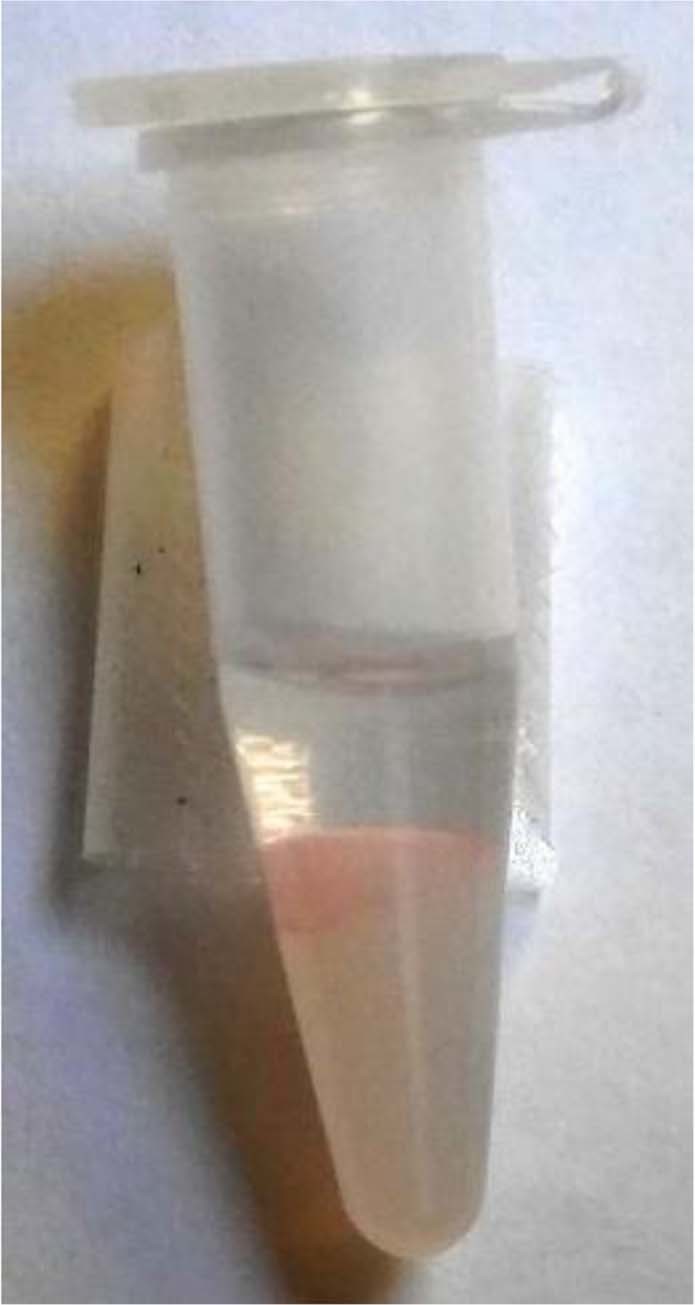
Partitioning of bacteriorhodopsin. A purple layer can be seen at the interface between the upper decanol phase and the lower water phase. The water phase is turbid from the addition of DS and DDAB, to help solubilize the bacteriorhodopsin and slightly purple from the protein itself.

Bovine serum albumin (BSA), *Taq* DNA polymerase (Tpol), and bacteriorhodopsin were tested for their ability to partition into decanol. BSA did not enter the decanol phase, even when 5 mM of PTAs were added. Tpol however did seem to enter the decanol to a small degree, even without PTAs present (Fig. 4). About 10% of the Tpol entered the decanol phase with no PTA or 5 mM of both DDAB and DS. Retention of secondary structure was evaluated in the water and decanol phases for Tpol, using circular dichroism. No alpha helical character was seen in the spectrum. We hypothesize this is due to the protein essentially turning inside out to enter the oil phase. Bacteriorhodopsin a membrane protein was found to only be soluble in amphiphilic systems; in our partitioning experiments it aggregated at the interface between the water and oil phase, perhaps also in colloidal structures within the water phase, seen as slightly purple turbidity (Fig. 5).

These proteins have very different sources and functions which could explain the difference in partitioning for each. BSA is used to transport hydrophobic material through the blood stream, and is therefore very water soluble, with a GRAVY score of about −0.5. Tpol is also water soluble, however it is known to be stabilized by a reduced entropic penalty to folding as opposed to non-covalent interactions, giving it thermostable behavior^20^. The GRAVY score for Tpol is about −0.25, making it more hydrophobic than BSA. Membrane proteins often have GRAVY scores above 0, for example bacteriorhodopsin has a score of 0.73. Interestingly the high hydrophobicity did not result in partitioning into the decanol. It was insoluble in both the water and decanol phases, appearing as crystals. The addition of PTAs allowed it to solubilize in perhaps an emulsion or vesicle phase. This result is not surprising as this membrane protien is know to be amphiphilic, interacting with the membrane bilayer at both the surface and interior.

### Other solvents

Decanol was originally chosen as it has been used previously to make cell models in oil, however it is clear that each solvent will have its own partitioning coefficients. Hexane, which is perhaps the best model for the low temperature ethane and methane liquid found on Titan, was tested and was not successful at partitioning any of the amino acids, likely do to its highly non-polar nature. Chloroform was challenging to measure as it absorbs strongly in the low UV ( < 240 nm) and was not compatible with HPLC for analysis.

## Conclusions

Most biomolecules are designed to function in aqueous phases, and therefore did not easily enter a decanol phase, even in the presence of phase transfer agents. Even the membrane protein, bacteriorhodopsin, which is not soluble in water, was unable to partition into the decanol layer. *Taq* DNA polymerase did have some solubility in decanol, and other proteins may as well, but it is unlikely that they would remain folded. The most successful biomolecule in this study was valinomycin, which did not need phase transfer agents. While valinomycin is a cyclic depsipeptide, not a traditional protein, this partitioning indicates that even polymers that look very similar to our own, could be used to generate life in a non-aqueous solvent.

There has been a surge of research focused on biopolymer modification that allows molecules to cross a membrane for pharmaceutical purposes, and these modifications hint to what requirements oil-based life may need. Modification of charged side chains with nonpolar moieties through esterification has been shown to increase cell permeability^21^. While N-acetylated polyproline has been shown to be somewhat soluble in octanol, modification of proline to an indole can also increase hydrophobicity of peptides by further burying the polar backbone and still allows helical conformation^22^, and that structural architectures like collagen can form in octanol with similar modification^23^ demonstrating the ability of peptide derivatives to form both secondary and quaternary structures in nonpolar solvents. These findings suggest that biopolymer derivatives would likely be more successful than their water-evolved counterparts.

The amino acids tested relied on phase transfer agents to partition into the decanol phase, and even then, only a small portion transferred well into decanol. Phenylalanine was chosen as it has a very hydrophobic side chain, with limited water solubility. We recognize that it is not generally thought that phenylalanine was part of the reduced amino acid alphabet on early Earth for the origin of life. However, in environments as are expected on Titan, molecules like phenyl groups are much more likely and could contribute to functional biopolymers. However, based on these findings, it is likely that a less charged monomeric form would be necessary as a precursor for nonpolar biopolymers.

Finally, we conclude that while extant biomolecules in the presence of phase transfer agents are not ideal for decanol-based life, the results shown here indicate that solubility in decanol even when water is present can be achieved, and future work should focus on the mechanisms that allow folding and function within non-aqueous phases to better understand changes in non-covalent interactions when water is not present.

## Methods

### Materials

Glycylglycine, decanol, and valinomycin were obtained from Acros Organics. Glycylglycylglycine and didodecyldimethyl ammonium bromide (DDAB) were purchased from Alfa Aesar. Glycine and *Taq* DNA polymerase were purchased from Fisher Scientific. Decyl sulfate was purchased from Oakwood Chemical, USA. Phenylalanine, glutamic acid, bovine serum albumin, and bacteriorhodopsin came from Sigma.

### Partitioning

Biomolecules were dissolved in ultrapure water (Millipore EMD, USA), containing decylsulfate and/or DDAB when noted. DDAB was not very soluble in the water phase even at 20 mM concentrations, but was sonicated with a probe sonicator (Branson Ultrasonics, USA) for a few seconds to increase the rate of dissolution. Final concentrations of phase transfer agents (PTAs) and amino acids were generally 10 mM unless noted otherwise for specific experiments. Proteins were generally diluted until their 280 nm absorbance was less than or equal to 1 AU.

Equal volumes of water phase (containing the biomolecule and PTA, if added) and decanol were mixed, vortexed for 30 s, then centrifuged for 3 minutes with a mini centrifuge. These samples were prepared in triplicate. Both decanol and aqueous phase were analyzed for biomolecules. Phenylalanine and proteins were analyzed using UV-vis (Hewlett-Packard 8453). Valinomycin and some glycine samples were analyzed using LC-MS (Ultimate 3000 LC with Velos Pro MS, Thermo Scientific). Glycine, polyglycine, and glutamic acid were analyzed using HPLC with charged aerosol detection (Ultimate 3000 with Corona CAD, Thermo Scientific).

### LC analysis

HPLC was performed with Cosmosil HILiC column (2.5 µm particle size, 3 × 100 mm; Nacalai USA) at 30 °C with a 1.0 mL/min flow rate, and isocratic elution of 25% 20 mM ammonium acetate, pH 7.5 and 75% acetonitrile (Optima, LC-MS purity, Fisher Scientific). Injection volume was 10 µL. For mass spectra collection, HESI heater temp was 50 °C, with a 4.5 kV spray, and 250 °C capillary temperature in positive ion mode.

## Supplementary information

Suppmentary information
