# Supplementary material for: Partitioning of amino acids and proteins into decanol using phase transfer agents towards understanding life in non-polar liquids
Source: Sci Rep. 2019 Nov 28;9:17750. doi: 10.1038/s41598-019-54322-8 (PMC6882823; doi:10.1038/s41598-019-54322-8)
Supplement: Supplementary file 1 — Suppmentary information [file 41598_2019_54322_MOESM1_ESM.docx]

Supporting information for

**Partitioning of amino acids and proteins into decanol using phase transfer agents towards understanding life in non-polar liquids**

Brooke Thompson, Kayla Burt, Andrew Lee, Kyle Lingard, Sarah E. Maurer*

Department of Chemistry and Biochemistry, Central Connecticut State University, New Britain CT 06050

Figure S1. UV 210 nm peak area of decanol phase after partitioning with three different ion conditions in the aqueous phase: 100 mM Au^+^, 100 mM K^+^, no added ions to ultra-pure water. Error bars represent deviations of three independently prepared partitioning experiments. No peaks were observed in the water phase.

Figure S2. Example chromatograph from UV detector at 210 nm. Partitioning of valinomycin into decanol. Blue line represents decanol phase of decanol/KCl partitioning. Black dotted line represents decanol phase of decanol/ultra-pure water partitioning. orange line represents water phase of decanol/KCl water partitioning.

Figure S3. Chromatograph of 1128.5-1129.5 m/z. Partitioning of valinomycin between decanol (blue) and aqueous 100 mM K^+^ (orange) phase. Grey line represents decanol phase of a separate decanol/ultra-pure water partitioning.

| A | B |
| --- | --- |
| Figure S4. Valinomycin mass spectra at 1 min retention time of decanol phase from mixture of decanol and (A) 100 mM AuCl or (B) 100 mM KCl . The base peak is 1128.87 Da. Other masses are 1111.82 ([M]^+^), 1333.78, 1149.76, and 1156.86. Samples are shown as two replicates of the partitioning. The base peak is likely [M+NH_4_]^+^ as our mobile phase contained ammonium acetate. | |

Figure S5. Valinomycin mass spectra at 1 min retention time of water phase from mixture of decanol and ultra-pure water.

Figure S6. Valinomycin mass spectra at 1 min retention time of decanol from mixture of decanol and ultra-pure water. Base peak is 1149.80 Da.
